# Novel Molecular Markers in Glioblastoma—Benefits of Liquid Biopsy

**DOI:** 10.3390/ijms21207522

**Published:** 2020-10-12

**Authors:** Zsuzsanna Birkó, Bálint Nagy, Álmos Klekner, József Virga

**Affiliations:** 1 Department of Human Genetics, Faculty of Medicine, University of Debrecen, 4032 Debrecen, Hungary; nagy.balint@med.unideb.hu; 2 Department of Neurosurgery, Faculty of Medicine, University of Debrecen, 4032 Debrecen, Hungary; klekner.almos@med.unideb.hu; 3 Department of Oncology, Faculty of Medicine, University of Debrecen, 4032 Debrecen, Hungary; virga.jozsef@med.unideb.hu

**Keywords:** liquid biopsy, circulating cell-free nucleic acids, glioblastoma, prognosis, integrated diagnostics

## Abstract

Glioblastoma is a primary Central Nervous System (CNS) malignancy with poor survival. Treatment options are scarce and despite the extremely heterogeneous nature of the disease, clinicians lack prognostic and predictive markers to characterize patients with different outcomes. Certain immunohistochemistry, FISH, or PCR-based molecular markers, including isocitrate dehydrogenase1/2 (IDH1/2) mutations, epidermal growth factor receptor variant III (EGFRvIII) mutation, vascular endothelial growth factor overexpression (VEGF) overexpression, or (O6-Methylguanine-DNA methyltransferase promoter) MGMT promoter methylation status, are well-described; however, their clinical usefulness and accuracy is limited, and tumor tissue samples are always necessary. Liquid biopsy is a developing field of diagnostics and patient follow up in multiple types of cancer. Fragments of circulating nucleic acids are collected in various forms from different bodily fluids, including serum, urine, or cerebrospinal fluid in order to measure the quality and quantity of these markers. Multiple types of nucleic acids can be analyzed using liquid biopsy. Circulating cell-free DNA, mitochondrial DNA, or the more stable long and small non-coding RNAs, circular RNAs, or microRNAs can be identified and measured by novel PCR and next-generation sequencing-based methods. These markers can be used to detect the previously described alterations in a minimally invasive method. These markers can be used to differentiate patients with poor or better prognosis, or to identify patients who do not respond to therapy. Liquid biopsy can be used to detect recurrent disease, often earlier than using imaging modalities. Liquid biopsy is a rapidly developing field, and similarly to other types of cancer, measuring circulating tumor-derived nucleic acids from biological fluid samples could be the future of differential diagnostics, patient stratification, and follow up in the future in glioblastoma as well.

## 1. Glioblastoma—Background

Glioblastoma (GBM) is a devastating primary central nervous system malignancy. Fighting the disease is challenging both for patients and the health care system. Survival is poor and treatment options are limited [1,2,3]. GBM belongs to the diffusely growing gliomas of the Central Nervous System (CNS), a heterogeneous group of tumors that are of glial, most commonly astrocytic or oligodendroglial origin [4,5]. This spectrum of diseases is characterized by their growth pattern, namely that the tumor cells aggressively invade the neighboring brain parenchyma, resulting in an extensive peritumoral infiltration. The invading cells leave the original tumor mass behind and travel to relative great distances in the brain, which result in the limitation of surgical resection, and later on, these cells become the source of tumor recurrence [6]. Despite the recent advances in the understanding of the disease—such as the development of molecular subgroups, characterization of common mutations, mapping of signaling pathways that lead to increased invasiveness, identifying molecules that contribute to glioma invasion—clinicians have yet failed to see any improvement in treatment options and patient outcomes [7,8]. GBM is characterized by a very extensive intratumoral heterogeneity, which further hinders effective treatment [9]. Furthermore, patients with seemingly similar clinical or histopathological features have very different response rates to the same treatment modality and often have very different survival [7,10]. More importantly, clinicians have no way to identify those patients who respond to standard therapeutic modalities or those who would have a more aggressive form of the tumor [11]. As of today, only some clinical factors have been identified with some correlation to patient outcomes, and the number of molecular markers that can be used as prognostic or predictive marker in glioblastoma is very limited.

## 2. Molecular Markers in Glioblastoma

Isocitrate dehydrogenase (IDH) mutations, (O6-Methylguanine-DNA methyltransferase) MGMT promoter methylation, (epidermal growth factor receptor) EGFR amplification/mutations, and vascular endothelial growth factor (VEGF) overexpression have been linked to be correlated with patient outcomes or treatment efficacy before.

IDH1/2 mutations are now part of the routine diagnostic pipeline, and bear not only diagnostic but prognostic relevance as well, as IDH mutant glioblastomas have better prognosis compared to IDH wild type [12]. IDH mutation is currently thought to be involved in gliomagenesis through the accumulation of oncometabolite 2-hydroxyglutarate, which is believed to lead progenitor cells into gliomas via multiple processes. IDH mutations are present in only about 10% of glioblastomas, but when present, they indicate better survival [13]. MGMT promoter methylation is a predictive marker of response to alkylating agent temozolomide (TMZ), a routinely used chemotherapeutic agent in glioblastoma treatment [14]. Translation of the MGMT gene results in a DNA repair enzyme, and hypermethylation of the MGMT promoter region results in the silencing of the gene, thus allowing TMZ to exhibit its therapeutic effect [15]. Overexpression and mutations (especially EGFRvIII mutation) is common in glioblastomas and are known to contribute to glioma invasiveness [16]. In many other types of cancers, the inhibition EGFR signaling was found to be an effective method to fight cancer; however, its amplification in GBM have failed so far [17]. The EGFRvIII mutation combined with other known factors (e.g., Ki67<20%, phosphatase and tensin homolog (PTEN) wild type, or MGMT hypermethylation could indicate better prognosis, and EGFRvIII positive tumors are candidates for vaccine-based novel experimental therapies [18]. VEGF is overexpressed in gliomas and is currently the only molecule suitable for targeted therapy in (recurrent) glioma. Bevacizumab, a VEGF inhibitor, was found to increase time to second progression in patients who were previously treated with temozolomide [19].

The above-mentioned markers are identified by immunohistochemistry, FISH, or PCR-based methods. They are routinely available in clinical practice; however, these methods always require tumor samples, and they cannot be used in real-time monitoring of treatment response, in screening for tumor recurrence, or in differential diagnosis. There is a dire need of novel prognostic and predictive markers in glioblastoma treatment, which could signal early tumor recurrence or ineffective treatment [20]. In other types of cancer, liquid biopsy methods have been found to correlate with treatment response even after a single treatment [21]. Liquid biopsy is a minimally invasive or non-invasive method, when blood, urine, saliva, or other bodily fluid samples are used in order to identify or monitor a disease, usually detecting small molecules of nucleic-acid fragments in the biological fluid. As glioblastoma is a tumor of the CNS, even histologic sampling could be a high-risk procedure, and treatment decisions today are based on analyses of the tissue samples. In multiple other types of cancer, however, liquid biopsies are now a standard of care in the diagnostic pipeline or therapeutic planning [22,23].

## 3. Circulating Tumor Nucleic Acids

**Figure 1:**
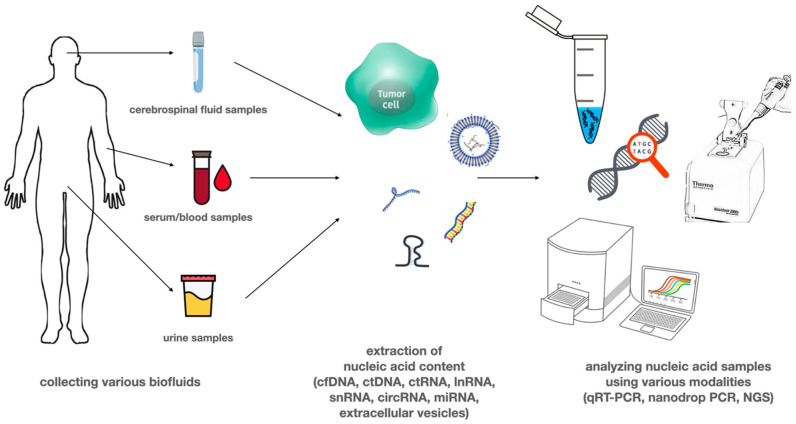
Liquid biopsy samples used in glioblastoma research. Liquid biopsy samples can be collected from any bodily fluid, including blood, urine, saliva, bronchial fluid, ascites, or cerebrospinal fluid (CSF). Due to disease characteristics, serum, CSF, and urine are the most commonly used in glioblastoma. Samples contain circulating tumor cells and other forms of nucleic acid, which are then used for quantitative and qualitative analysis.

Several tumor-derived circulating nucleic acids (e.g., ctDNA, cmtDNA, mRNA, non-coding RNAs including miRNAs, long non-coding RNAs) can be detected from blood or other types of body fluids like urine, cerebrospinal fluid (CSF), saliva, pleural fluid, and ascites [21]. These different types of potential biomarkers in the blood can be present in cell-free forms, attached to lipid or protein structures, or delivered by circulating extracellular vesicles (EVs) or platelets [24]. Figure 1 shows liquid biopsy specimens most commonly used in glioblastoma research. Serum and urine are most commonly used, as samples are easy to collect; however, the amount of circulating tumor-derived nucleic acid or the number of circulating tumor cells is low due to the biological properties of glioblastoma. CSF has a higher amount of nucleic acid than CTC; however, it was found that lumbar puncture is less likely to yield good results compared to suboccipital cisternal punction, which is a high risk procedure [21,22,23,24].

## 4. Circulating DNA

Cell-free DNA (cfDNA) as a double-stranded, fragmented DNA that is approximately 150 to 200 base pairs in length, corresponding to nucleosome-associated DNA, can be released by cells under physiological and pathological conditions as well. It is suggested that cfDNA could be derived from apoptotic or necrotic cells [25], rapidly dividing cells or CTCs [26]. Additionally, macrophages may actively release cfDNA into the serum [27] as well. Molecules of cfDNA are rapidly cleared by phagocytosis, and as a consequence, levels of cfDNA are typically low in healthy individuals, approximately 10 to 15 ng/ml in plasma [28]. In cancer patients, cfDNA that is released from tumor cells is called circulating tumor DNA (ctDNA) and gives only a fraction of overall cfDNA that can vary over a wide range, from less than 0.1% to more than 90% [29]. Generally, ctDNA is present in increased concentrations in various liquid biopsy samples, for example blood samples of cancer patients, including glioblastoma, compared to control individuals. Bettegowda et al. analyzing the ctDNA obtained from 640 patients, found a correlation between the concentration of tumor-specific variants detected in plasma and survival for those with metastatic disease burden [29,30]. According to some studies, in patients who respond to therapy, ctDNA levels of tumor-specific variants drop dramatically within 1 to 2 weeks after starting treatment. Tumor-specific mutations of ctDNA can be an important source of information about the given type of cancer. Analyzing the mutations carried by the ctDNA can be an effective tool for early detection; furthermore, it can help in the identification of the molecular alterations that lead to resistance to a particular therapy [31]. Resistant subclones can be present at the same time in the lesion, and as a consequence, a single-lesion tumor biopsy does not reflect the existing molecular heterogeneity of the tumor [32].

Patients with different tumor types show considerable variation in the frequency of detectable ctDNA. In glioblastoma, the concentration of cancer cell derived ctDNA in plasma is low compared to that of other cancer types, which could be mainly the consequence of the presence of the blood–brain barrier (BBB) [29]. There are several challenges associated with ctDNA analysis in glioblastoma. Besides the generally existing problems, such as short half-life (<1.5 h) of ctDNA fragments, distinguish mutant form wild-type alleles, or the development of thresholds for mutations, the main problem is the low amount of ctDNA in the samples [33]. Applying new technologies such as droplet-based digital PCR (ddPCR) and optimized forms of next-generation sequencing (NGS) techniques have improved the sensitivity and specificity for the detection of cfDNA mutations [34]. In the future, both methods can be employed in clinical assays depending on the context. While NGS allows exploring a wide range of possible new mutations in a particular ctDNA fragment, with the help of ddPCR, known specific mutations can be tested. The ddPCR method is also useful in confirming mutations identified with next-generation sequencing [35,36].

An initial study conducted by Bettegowda et al. detected mutations, such as those present in IDH1, TP53, EGFR, and PTEN, were limited to a subset (10%) of patients with glioblastoma [29]. On the other hand, a more recent study performed by Piccioni et al. applying a highly sensitive and specific NGS panel, detected ctDNA mutations in 55% of plasma samples collected from 222 GBM patients. Some of the alterations identified in this patient cohort including *BRAF/IDH1/IDH2* mutations, *ERBB2/MET/EGFR/PDGFRA* amplifications, and mutations in DNA damage repair genes show potential for molecular targeted therapies [37]. The option to detect these genomic alterations through ctDNA analysis might provide a comprehensive view of the glioblastoma genome including ctDNA derived from different tumor regions. Altered epigenetic modifications have also been identified in plasma and CSF fluid samples of GBM patients as well. The change in DNA methylation status, for example, can indicate acquired resistance to a certain treatment, therefore monitoring those alterations can help in choosing a personalized therapy [38]. Lavon et al. examined the *MGMT* promoter methylation in tissues and the corresponding serum of patients with different grades of glioma (grade II–IV). The overall sensitivity of the test for *MGMT* gene promoter methylation in serum samples was 51% and specificity was 100% [39]. Due to the presence of the blood–brain barrier, the cerebrospinal fluid (CSF) should contain higher levels of circulating tumor DNA, particularly for primary tumors of the nervous system, such as gliomas. Wang et al. analyzed the MGMT gene promoter methylation in serum and CSF samples of patients with different grades of glioma. Among all the CSF and blood samples, 21.3% of the blood samples and 33.3% of the CSF samples were positive for *MGMT* promoter methylation suggesting that the analysis of CSF samples may be preferable compared to serum samples [40]. However, lumbar puncture is contraindicated in patients who have an increased intracranial pressure due to an intracranial mass. Besides the alteration of MGMT promoter methylation, according to Chen et al., glioblastoma patients presented lower levels of Alu methylation than the controls in plasma samples. A significant correlation between survival and methylation levels could be observed in this analysis, as the longer the overall survival of the patient was, the higher the levels of Alu methylation were in these patients [41].

Besides the ctDNA, several studies have demonstrated that alterations in mtDNA content play a role in cancer development as well [42]. According to a study performed by Cormio et al., alterations of mtDNA content occur early in premalignant lesions [43]. Zhang et al. has also demonstrated that higher peripheral leukocyte mtDNA content is associated with increased risk of glioma [44]. Furthermore, Chen et al. using a real-time PCR-based method analyzed the correlation of mtDNA levels with overall survival (OS) and progression-free survival (PFS) of glioma patients after tumor resection. They concluded that patients with high mtDNA content showed significantly poorer OS and PFS than those with low mtDNA. Additionally, the elevated mtDNA level was associated with lower NK-cell frequency and higher IL-2 and TNF-a concentrations suggesting that high mtDNA content may contribute to the progression of gliomas, possibly through the abnormal alteration of immune functions [45]. Mair et al. investigated the levels of tmtDNA and ctDNA in different rat patient-derived orthotopic xenograft (PDOX) models of GBM, in an experiment using tumor samples taken from GBM patients. In this study, the detection rate for tmtDNA was 82%, while ctDNA detection rate was only 24% in plasma samples. Furthermore, tmtDNA can be identified in 60% of urine samples, while ctDNA was not detectable in urine [46]. This study shows the potential of tmtDNA in the early diagnosis of GBM or in monitoring its recurrence.

## 5. Circulating RNA

Circulating tumor RNA (ctRNA) include mRNAs, long non-coding RNAs (lnRNAs), and small non-coding RNAs (snRNAs). Among small ncRNAs, we can find for example microRNAs (miRNAs) and circular RNAs (circRNAs). Circulating RNAs are present in a highly stable form in plasma, perhaps as a consequence of their association with subcellular particles or exosome packaging [47].

miRNAs are one of the promising biomarkers for cancer diagnosis. miRNAs as small, usually 19–25 base-pair long ncRNAs, are involved in most physiological and pathological processes, such as apoptosis, proliferation, differentiation, migration, or invasion via regulating post transcriptional gene expression [48]. miRNAs are the most abundant circulating free molecules in blood; moreover, they can be detected in several other types of bodily fluids like urine, saliva, and CSF [49]. While it appears that, in case of ctDNA, the CSF is a better source for analysis in brain tumors such as glioblastoma, there is no consensus on the optimal source of miRNA. A meta-analysis performed by Qu et al. concluded that panels containing miR-21, regardless of source, may be more specific for glioma [50]. mir-21 is upregulated in GBM patients and is associated with poorer overall survival and tumor grade [51]. Furthermore, its levels decreased after chemo-radiation, so it could be used as a biomarker of therapeutic response. Besides the significant upregulation of miR-21, Wang et al. detected the downregulation of miR-128 and miR-342, correlating with glioma grades in plasma and tissue samples of glioblastoma patients compared with healthy controls [52]. In contrast to miR-21 levels, miR-128 and miR-342 concentration increased after surgery and chemo-radiation, suggesting their use in monitoring the treatment response as well [52] (Table 1). Furthermore, besides the above-mentioned miRNAs, the expression level of several other miRNAs have been found to be altered by other studies. Increased levels of miR-221, miR-222, miR-210, miR-182, or miR-454 can be observed in serum samples of glioblastoma patients and are associated with tumor progression and low survival rates [53] (Table 1).

Along with miRNAs, the circulating lncRNAs have the potential to be diagnostic and prognostic biomarkers in glioblastoma as well. lncRNAs can vary in length from 200 nucleotides to 100 kilobases, similarly to mRNAs with 5’m7G caps and 3’ poly(A) in tails but lack significant protein-coding capacity [54]. LncRNAs compared with the protein-coding genes have a more tissue specific expression patterns, and their expression is highly associated with their biological function and tumor status [55]. lnRNAs take part in onset and progression of cancer malignancy, such as proliferation, angiogenesis, and drug resistance via deregulation of several signaling pathway [56]. HOX Transcript Antisense RNA (HOTAIR) was detected as a negative prognostic factor in several cancers, including glioblastoma, and to be significantly associated with overall survival [57,58]. Jie et al. analyzed six oncogenic or tumor suppressor lncRNAs (Colorectal Neoplasia Differentially Expressed (CRNDE), Growth Arrest Specific 5, H19 (GAS5), H19, (HOX Transcript Antisense RNA) HOTAIR, Metastasis Associated Lung Adenocarcinoma Transcript 1 (MALAT1), and Taurine Up-Regulated 1 (TUG1) in the serum from 106 patients with primary glioblastoma. HOTAIR and GAS5 levels were associated with 2-year overall survival and disease-free survival in patients with glioblastoma. According to these results, high HOTAIR and low GAS5 levels had worse survival rates compared to patients with low HOTAIR and high GAS5 levels [59] (Table 1). Furthermore, high levels of HOTAIR were associated with a 1.82-fold increase in the likelihood of recurrence or progression, while high levels of GAS5 were associated with a 54% decrease in the likelihood of recurrence or progression [59]. It is important to mention that lncRNAs and miRNAs form a complex regulatory network in mRNA stabilization and degradation. In addition, miRNAs are more likely to function as a bridge between non-coding RNAs and mRNAs [60].

In addition to miRNAs and lncRNAs, the highly stable, evolutionary-conserved, and tissue-specific circular RNAs are other key elements of tumorigenesis and development. Circular RNAs, consisting of a covalently closed-loop structure, have been recently rediscovered as a small non-coding type of RNA without 5’ cap 3’ poly-A tail that makes them resistant to exonuclease [50]. circRNAs are mostly noncoding, but a few can be translated into polypeptides [61]. There are different types of circular RNAs such as exonic circRNAs (ecircRNAs), intronic RNAs (ciRNAs), exon intron circRNAs (EIciRNAs), intergenic circRNAs [62]. circRNAs can be involved in cancer development as oncogenes or tumor suppressors on the base of several different mechanisms: acting as miRNA sponges [63], regulating gene expression [64], interacting with RNA-binding proteins (RBPs), or to be involved in RNA and protein transfer and storage [65]. According to the studies, circRNAs that take part in glioma progression mainly act as miRNAs sponges [66]. Circular RNAs are more enriched in neuronal tissues compared with other tissues, partly due to the abundance of specific genes promoting circularization. This fact suggests that aberrant expression of circRNAs is closely related to diseases of the nervous system, including gliomas [67]. Gene Ontology (GO) analysis showed that downregulated circRNAs were significantly associated with protein binding and the large RNA–protein complexes might regulate the pool of RNA-binding proteins. Furthermore, the upregulated circRNAs are included G0–G1 transition, manganese ion transport, or store-operated calcium channel activity, and these processes could be involved in the development of gliomas [68]. circRNAs are present in blood and other bodily fluids; however, to date, the role of dysregulation of circRNAs in glioma tumorigenesis was barely analyzed in tissue and exosome samples of GBM patients. Wang et al. reported that downregulation of circ_0001649 that normally facilitates apoptosis by regulating Bcl-2/caspase-3 pathway was linked to larger tumor size and advanced WHO grade, indicating circ_0001649 may be an independent prognostic marker in GBM recurrence monitoring after surgery [69]. The study of Zhu et al. demonstrated that the upregulation of circBRAF can be an independent predictive factor with good progression-free survival and overall survival in glioma patients by Cox analysis [68]. Furthermore, circ_0034642, circ_0074362, circ_ITCH, circHIPK3, and circCPA4 linked to clinical severity and poor prognosis in patients with glioma [70] (Table 1). In the future, identification of circRNAs in samples originating from different bodily fluids would promote circRNAs to act as valuable biomarkers for diagnosis, prognosis, and monitoring of glioma.

## 6. Extracellular Vesicles

Extracellular vesicles are a promising new source of macromolecules gained by liquid biopsy for minimally or non-invasive diagnosis and monitoring cancer in humans. Exosomes that are able to cross the blood–brain barrier (BBB) are small (40–150 nm in diameter), membrane-enclosed extracellular vesicles produced and secreted by many different cell types, and are present in different type of the bodily fluids such as blood, cerebrospinal fluid (CSF), urine, or saliva [71]. Exosomes have a diverse molecular composition including nucleic acids, proteins, lipids, and metabolites that are protected via the double membrane layers from proteases, nucleases, and other degrading factors [71]. Studies have shown that some molecules are enriched in exosomes, while others are not present, depending on the cell type, and this can vary in response to the specific stimulus. Following secretion, EVs can interact with neighboring or distant cells via direct membrane fusion, ligand-receptor interaction, or endocytosis, modulating the activity of the recipient cells. In this context, several studies have demonstrated that exosomes play important role in cell-to-cell communication and are involved in cancer development, transmitting oncogenic signals to recipient cells, promoting their tumorigenic activities. Related to that, tumor-derived exosomes can create a tumor-permissive microenvironment via changing the behavior of surrounding stromal cells, which in response, would secrete exosomes as well, promoting proliferation, invasion, angiogenesis, metastases, immune-escape, or treatment-resistance further [72]. Several evidence from this field suggests that exosomal bioactive molecules be transferred between different cell populations, and that they participate in initiation and progression of glioma. The study from Osti et al. revealed that the concentration of EVs was increased in GBM patients compared with healthy controls, furthermore the EV concentrations showed a correlation with tumor recurrence [73]. Additionally, the proteomic profiling of these EVs showed a glioblastoma-specific pattern with EGFR amplifications, PTEN deletions, IDH1/2, and TP53 mutations [73]. In another study, it was found that syndecan-1 present in plasma EVs can be useful to distinguish low-grade from high-grade glioma with a sensitivity of 71% and a specificity of 80% [74]. Certain miRNAs, as it was demonstrated for example by Ebrahimkhani et al., are selectively packaged into exosomes regulating cell proliferation in glioma. This research group used a panel of seven exosomal miRNAs—miR-182-5p, miR-328-3p, miR-339-5p, miR-340-5p, miR-485-3p, miR-486-5p, and miR-543—to differentiate GBM patients from healthy controls with an accuracy rate of 91.7% [75]. Santangelo et al. detected the upregulation of three miRNAs, miRNA-21, miR-222, and miR-124-3p in serum samples of glioma patients, and the combination can help differentiating high-grade gliomas from brain metastases of other tumors [76]. Emerging evidence suggests that EVs released from TMZ-resistant glioma cells are able to transfer drug resistance to recipients’ cells, which are still TMZ-sensitive via their molecular cargos. Zhang et al. reported that lncRNA SBF2-AS1 in upregulated form is associated with TMZ resistance and could help the spread the TMZ resistance to the TMZ-sensitive cells further by exosomal transport [77]. Besides promoting the spread of chemoresistance, macromolecules within the EVs can influence the efficiency of radiotherapy as well. Zhao et al. demonstrated that circRNA-ATP8B4 derived from EVs of radioresistant GBM cells can spread glioma radio-resistance by sponging miR-766 [78]. Considering the importance of EVs in tumor evolution and in treatment response rates, several studies are related to modulate exosome production or block exosome uptake pathways. The concept of reducing circulating cancer-specific exosomes could be a promising treatment for cancer patients [79]. Marleau et al. found that applying a plasmapheresis platform called Aethlon ADAPTTM (adaptive dialysis-like affinity platform technology) can incorporate diverse affinity agents for capturing cancer-specific exosomes on the basis of displaying surface proteins or glycoproteins [80].

**Table 1:** Various miRNAs, lncRNAs, circRNAs as diagnostic/prognostic biomarkers in glioblastoma (GBM).

miRNA	Expression in GBM	Effect of Altered Expression	Source of Samples	
miR-21	Upregulation	high levels are associated with poor prognosis, levels drop after chemoirradiation	EVs	[50]
miR-128	Downregulation	downregulated in glioma, levels increase after surgery and chemoirradiation	plasma and tissue	[52]
miR-342	Downregulation	downregulated in glioma, levels increase after surgery and chemoirradiation	plasma and tissue	[52]
miR-221	Upregulation	increased levels are associated with tumor prognosis and low survival rates	serum	[53]
miR-210	Upregulation	increased levels are associated with tumor prognosis and low survival rates	serum	[53]
miR-182	Upregulation	increased levels are associated with tumor prognosis and low survival rates	serum	[53]
miR-454	Upregulation	increased levels are associated with tumor prognosis and low survival rates	serum	[53]
**lncRNA**				
HOTAIR	Upregulation	elevated levels are associated with poor prognosis, early tumor recurrence	serum	[57], [58]
GAS5	Upregulation	elevated levels are associated with better prognosis and decreased chance of recurrence	serum	[59]
SBF2-AS1 lncRNA	EVs	associated with TMZ resistance	EVs	[77]
**circRNA**				
circ_0001649	Downregulation	associated with larger tumor size and advanced WHO grade	tissue	[68]
circ_BRAF	Upregulation	associated with better progression free and overall survival	tissue	[68]
circ_0034642	Upregulation	associated with poor prognosis	tissue	[70]
circ_0074362	Upregulation	associated with poor prognosis	tissue	[70]
circ_ ITCH	Upregulation	associated with poor prognosis	tissue	[70]
circHIPK3	Upregulation	associated with poor prognosis	tissue	[70]
circCPA4	Upregulation	associated with poor prognosis	tissue	[70]
ATP8B4 circRNA	Upregulation	associated with insensitivity to radiotherapy	EVs	[78]

EV—Extracellular Vesicles; TMZ—Temozolomide; WHO—World Health Organization.

## 7. Circulating Tumor Cells

**Figure 2:**
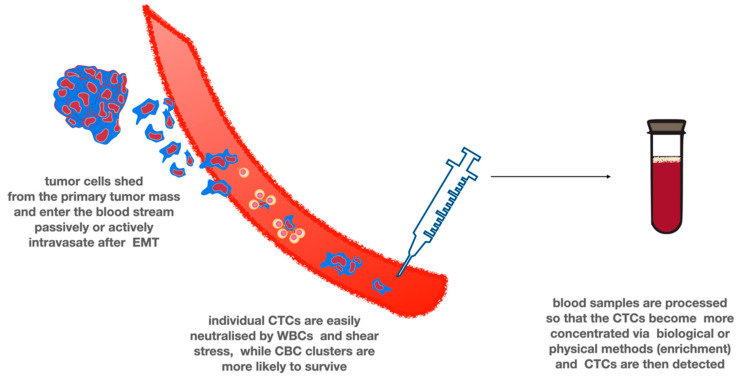
Circulating tumor cells. CTCs are cornerstone of liquid biopsies in many types of cancer. The tumor cells enter the blood stream either passively by shedding or actively after EMT. Most tumor cells quickly die due to the shear stress and as the result of the attack of white blood cells. However, forming clusters and interacting with platelets are protective from being cleared from the circulation. CTCs must be enriched prior to detection due to their low number in the circulation. Multiple techniques exist, using different biological or physical properties of CTCs.

Tumor cells leaving the primary tumor site and entering the circulation are termed circulating tumor cells (CTCs). CTCs as metastatic precursor cells that undergo epithelial-mesenchymal transition can be released as single cells or in homotypic or heterotypic clusters, which could have a higher metastatic inclination than single CTCs. (Figure 2) It is not known whether CTCs represent only subpopulations of the central tumor or rather the entire original tumor [74]. Extracranial metastasis of glioblastomas is a very rare event (0.4 to 0.5% of patients) affecting mostly the lungs and pleura, regional lymph nodes, bones, and liver [74]. Several reasons for the low rates of detectable metastasis, for example, might be the low survival rates of GBM patients, due to the presence of the BBB, which makes it more difficult for the cells to intravasate into the circulation, or it is possible that GBM cells require critical neural-specific growth factors that are absent outside the brain. Alternatively, immune-mediated suppression of GBM cells harboring epitopes that are usually masked by the blood–brain barrier could block the tumor cell growth outside the CNS [81]. CTCs are exceedingly rare with only 1 CTC per 10^9^ blood cells, and their isolation is difficult because of the complexity of the required techniques. CTCs have been detected and characterized in different tumor types, and their presence has been found to correlate with poor overall survival [74]. CTCs can be isolated based on different approaches. Negative-enrichment techniques are based on CTC size (CTCs are larger than normal blood cells) or other biophysical properties, while positive selection of CTCs can be achieved by the detection of specific tumor markers that are commonly expressed on the surface of these cells [53].

In the case of glioblastoma, CTCs were first identified by Muller at al. in 2014. This group detected fibrillary acid protein-positive (GFAP), CD45-negative CTCs in 20.6% of patients with glioblastoma and found to harbor specific aberrations of the primary tumor, including EGFR gene amplification, or chromosome insertions or deletions. Following surgery, no significant difference was observed in CTC counts before, during, or after surgery and no correlation between CTC counts and clinical outcome [81]. MacArthur et al. detected brain tumor cells in the peripheral blood by a telomerase promoter-based assay combined with a test for nestin expression as a glioma cell marker. The assay employees a telomerase-responsive adenoviral vector encoding a fluorescent reporter to detect hTERT-positive cells. With the help of this method, they managed to detect CTCs in eight out of 11 (72%) glioblastoma patients analyzed before radiotherapy and TMZ treatment, while the detection rate for postradiotherapy patients was one out of eight (8%) [82]. Sullivan et al. developed a “negative-depletion” CTC-iChip, which effectively removes leukocytes from blood samples, via magnetically tagged antibodies (CD16, CD45), yielding untagged and un-manipulated CTCs. In this study, 13 of 33 adult patients with glioblastoma harbored CTCs. CTCs were then characterized by using antibodies against SOX2, tubulin β-3, EGFR, A2B5, and c-MET representing all the molecular subtypes of GBM (proneural, neural, classical, and mesenchymal) [83]. Molecular characterization of expression markers within individual GBM CTCs identified enrichment for mesenchymal transcripts and reduction of neural differentiation markers. It can be established that the number of studies showing the detection of CTCs in GBM is still limited, and the diagnostic, prognostic, predictive, and monitoring potential of CTCs in case of glioblastoma still need further investigation [74].

## 8. Summary

Despite recent developments in the understanding of the disease, glioblastoma is still a riddle that is difficult to break. Treatment options are very limited, most patients face poor prognosis, while clinicians struggle to identify patients who are resistant to standard treatment protocols.

In recent decades, the findings of glioma molecular tumor biology has led to major milestones, yet the long-awaited breakthrough has not yet arrived. Some research findings, such as those analyzing genetic mutations, have helped us support the differences among patients, resulting in the molecular subgroup classification of glioblastoma. Others, such as the IDH mutations, or the 1p/19q codeletion are included in the diagnostic testing of glial tumors. However, prognostic and predictive markers are still not routinely available. Some markers, including the MGMT promoter hypermethylation or IDH mutations are well-known, yet they are either rare (IDH mutations) or bear limited therapeutic consequences (MGMT promoter hypermethylation).

Liquid biopsy is an increasingly developing field of research and the number of cancers where liquid biopsy methods are routinely used in clinical practice is continuously evolving. Serum, urine, CSF, and other bodily fluid contain small nucleic-acid particles that are linked to prognosis or treatment response. A wide range of these particles have been studied in glioblastoma and are now considered as novel markers of glioblastoma prognosis or treatment response (see Table 1). Some of these could be useful in the diagnosis, others could be used in early follow up after surgery or radio-chemotherapy, and some could also be used to identify tumor recurrence or the development of therapeutic resistance. The benefits of using these markers over traditional markers include the minimal invasiveness of sampling; furthermore, the technical requirements of measuring levels of circulating nucleic-acid particles is becoming more and more available in clinical laboratory facilities as well. However, more research is needed to validate the clinical benefit of these promising markers.
